# Innate Defense Regulator Peptide 1018 in Wound Healing and Wound Infection

**DOI:** 10.1371/journal.pone.0039373

**Published:** 2012-08-06

**Authors:** Lars Steinstraesser, Tobias Hirsch, Matthias Schulte, Maximilian Kueckelhaus, Frank Jacobsen, Evgenija A. Mersch, Ingo Stricker, Nicole Afacan, Havard Jenssen, Robert E. W. Hancock, Jason Kindrachuk

**Affiliations:** 1 Department of Plastic Surgery, BG University Hospital Bergmannsheil, Ruhr University Bochum, Bochum, Germany; 2 Institut for Pathology, BG University Hospital Bergmannsheil, Ruhr University Bochum, Bochum, Germany; 3 Centre for Microbial Diseases and Immunity Research, University of British Columbia, Vancouver, British Columbia, Canada; 4 Department of Science, Systems, and Models, Roskilde University, Roskilde, Denmark; University Hospital Hamburg-Eppendorf, Germany

## Abstract

Innate defense regulators (IDRs) are synthetic immunomodulatory versions of natural host defense peptides (HDP). IDRs mediate protection against bacterial challenge in the absence of direct antimicrobial activity, representing a novel approach to anti-infective and anti-inflammatory therapy. Previously, we reported that IDR-1018 selectively induced chemokine responses and suppressed pro-inflammatory responses. As there has been an increasing appreciation for the ability of HDPs to modulate complex immune processes, including wound healing, we characterized the wound healing activities of IDR-1018 *in vitro*. Further, we investigated the efficacy of IDR-1018 in diabetic and non-diabetic wound healing models. In all experiments, IDR-1018 was compared to the human HDP LL-37 and HDP-derived wound healing peptide HB-107. IDR-1018 was significantly less cytotoxic *in vitro* as compared to either LL-37 or HB-107. Furthermore, administration of IDR-1018 resulted in a dose-dependent increase in fibroblast cellular respiration. *In vivo*, IDR-1018 demonstrated significantly accelerated wound healing in *S. aureus* infected porcine and non-diabetic but not in diabetic murine wounds. However, no significant differences in bacterial colonization were observed. Our investigation demonstrates that in addition to previously reported immunomodulatory activities IDR-1018 promotes wound healing independent of direct antibacterial activity. Interestingly, these effects were not observed in diabetic wounds. It is anticipated that the wound healing activities of IDR-1018 can be attributed to modulation of host immune pathways that are suppressed in diabetic wounds and provide further evidence of the multiple immunomodulatory activities of IDR-1018.

## Introduction

Cutaneous wound repair is a specialized, multifactorial process that involves a large number of factors that are involved in the regulation of this process [1]. Although initially characterized for their direct antimicrobial activities [2], [3], [4], there is an increasing appreciation for the immunomodulatory activities of cationic host defense peptides (HDPs; also termed antimicrobial peptides). HDPs are ubiquitous in nature and form central components of the innate immune system of eukaryotes. The immunomodulatory activities of such natural peptides, especially the human cathelicidin LL-37 and human β-defensin (hBD) -3, are extremely diverse and include, but are not limited to, the stimulation of epithelial cell migration, promotion of angiogenesis, and suppression of pro-inflammatory responses [3], [5], [6], [7], [8]. For example, the human cathelicidin peptide LL-37 attracts neutrophils, monocytes, mast cells, and T lymphocytes [9], [10], and also induces the production of neutrophil and monocyte chemoattractants by many cell types [3], [11], [12], [13], [14]. Recently, HDPs have also been implicated as regulators of cutaneous wound repair by regulating inflammation, angiogenesis, and extracellular tissue deposition and remodeling [3], [15], [16], [17], [18].

The highly diverse sequences and versatile antimicrobial and immunomodulatory activities of natural HDPs provides a wide range of templates for the design and development of synthetic peptides with activities tailored for clinical applications [3]. A number of peptides and peptidomimetic compounds are currently undergoing clinical trials; however, most HDP clinical trials are aimed at primarily at topical applications of peptides and are based on their direct microbicidal properties (reviewed in [3], [19], [20]).

It has been shown that the influence of HDPs on wound repair is not dependent on antimicrobial function and provides a potential novel clinical application for HDPs [21]. For example, HB-107, a synthetic HDP originally derived from the antimicrobial cecropin B, was shown to promote wound healing [21]. A new group of synthetic variants of HDPs, termed innate defense regulators (IDRs), which provide broad-spectrum protection against systemic infections with multidrug-resistant bacteria, have recently been described [22], [23], [24], [25]. IDR-1 and IDR 1002 confer protection against microbial challenges by enhancing innate immune defenses of the host while suppressing potentially harmful excessive inflammatory responses [22], [23], [24], [25]. Such selective enhancement of innate immunity represents a novel approach to anti-infective therapy with many advantages over directly microbicidal compounds [26], [27], [28]. Although many HDPs exert their direct antimicrobial activity through disruption of microbial membranes, IDRs appear to exert their immunomodulatory activities through non membrane-lytic mechanisms. Thus issues of hemolytic activity and cytotoxicity toward mammalian cells are minimized, as supported by the minimal toxicity of peptide IDR-1 compared with natural cathelicidins such as LL-37 [25], [28].

Despite the increasing interest in utilizing the immunomodulatory activities of HDPs and IDRs for clinical applications, investigations into optimizing and enhancing peptide immunomodulatory properties have thus far been limited. However, it is apparent that the ability to modulate chemotactic activity and chemokine induction are important aspects of the immunomodulatory activities shared by many natural HDPs. Recently, we screened a series of more than 100 distantly related peptide variants of bovine HDP Bac2A (unrelated by sequence to IDR-1, [22]) for improved immunomodulatory activity as assessed by chemokine production in human peripheral blood monocytic cells. The most active peptide observed was IDR-1018 (VRLIVAVRIWRR-NH_2_) which was shown to form an α-helix in neutral membranes [29].

In the current work, we have expanded on the characterization of the immunomodulatory activities of IDR-1018 and demonstrated that it also promotes wound healing activities. IDR-1018 promoted wound healing in normal and infected wounds, and its effects were favourably compared to those of naturally occurring LL-37 and synthetic HB-107 as prospective positive controls.

## Results

### Effects of IDR-1018 on cell viability and migration

**Figure 1:**
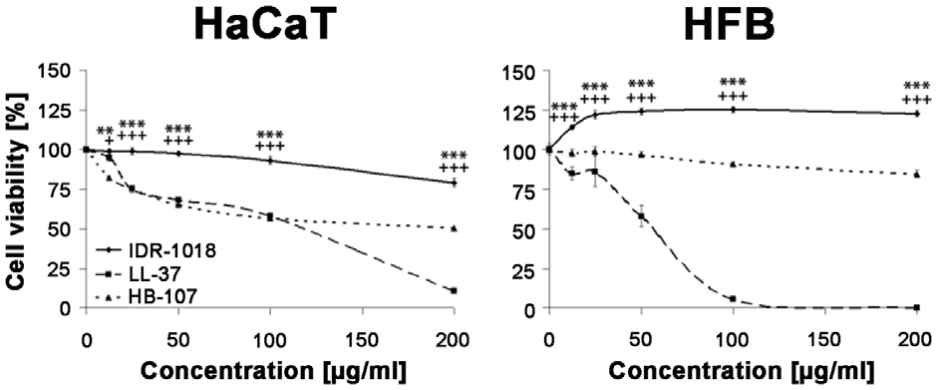
Lack of IDR-1018 toxicity. The effects of IDR-1018, LL-37 and HB-107 on viability/metabolic activity were determined using an MTT-Assay with HaCaT cells (left) and primary human fibroblasts (HFB; right) at the indicated concentrations. Cells were incubated with peptides for 24 hours. Standard deviation was calculated out of three independent experiments and assessed as SEM (Statistical significance IDR-1018 vs. LL-37: *  = p<0.05; **  = p<0.01; ***  = p<0.005; IDR-1018 vs. HB-107^+^ = p<0.05).

To investigate of effects of IDR-1018 on cell oxidative metabolism, an MTT-Assay was performed on HaCaT (immortalized human keratinocyte cell line) and primary human fibroblasts (HFB). In this assay, toxicity is assessed as residual metabolic activity through the production of MTT formazan crystals by mitochondrial dehydrogenases in living cells. The HDPs LL-37 and HB-107 served as peptide controls for naturally occurring and synthetic HDPs with wound healing activity [21], [30]. IDR-1018 treated HaCaT cells maintained cell viability better than those treated with LL-37 or HB-107 at all concentrations analyzed in this study (Fig. 1). In all samples, cell viability was affected in a concentration dependent manner. LL-37 showed a significant decrease in cell viability at higher concentrations (10% compared to untreated cells at 200 µg/ml (p<0.0001), consistent with literature data demonstrating that this peptide is cytotoxic at higher concentrations. Moreover, HB-107 exhibited a reduction in HaCaT cell viability of up to 50% compared to untreated cells (p<0.0001). In contrast, IDR-1018 administration had very little effect on the metabolic activity of HaCaT cells as compared to LL-37 and HB-107 (78% residual metabolic activity at 200 µg/ml; p<0.0001 compared to LL-37 and or p<0.001 compared to HB-107 or p = 0.0013 compared to untreated cells).

In HFB, incubation with LL-37 or HB-107 led to significant decreases in cell viability. At a concentration of 200 µg/ml, LL-37 led to a complete inhibition of cell metabolism (p<0.0001). Administration of HB-107 slightly decreased cell viability as compared to non-treated cells (p<0.0001). In contrast, IDR-1018 administration was completely non-cytotoxic and instead appeared to increase cell viability. Indeed, even low doses of IDR-1018 increased cell metabolic activity compared to untreated HFBs (25 µg/ml: 121% compared to untreated cells; p = 0.0185), or HFBs treated with other peptides, (p = 0.0004).

### Dose-dependent effect of IDR-1018 on wound healing

**Figure 2:**
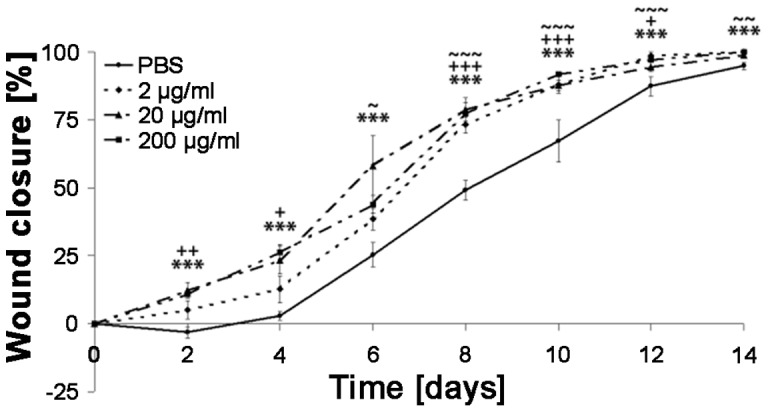
Efficacy in wound healing of IDR-1018 peptide in a murine dose-response model. Peptides were administered every 48 hours with the indicated concentrations of IDR-1018 or PBS (negative control). Re-epithelialization/wound closure was measured every second day. Standard deviation was assessed as SEM (Superscript symbols *, ^+^, and ^∼^ represent 15 µl doses of 3, 0.3 and 0.03 µg respectively; *,+,∼  = p<0.05; **,++,∼∼  = p<0.01; ***,+++,∼∼∼  = p<0.005 compared to vehicle control).

To analyze the effects of IDR-1018 in the complex process of wound healing we performed a dose response study using a splint-model in non-diabetic mice (n = 5 for each group). The application of IDR-1018 led to significant increase in wound closure in a dose-dependent manner as compared to vehicle control (Fig. 2). Treatment with the highest dose of IDR-1018 (200 µg/ml) resulted in the greatest acceleration of wound closure. This dosage of IDR-1018 also resulted in the most significant difference in wound closure as compared to vehicle control. Wound closure in IDR-1018 treated animals led to 14 28% smaller wound areas as compared to controls from days 2–8 (p<0.0001 to p = 0.0087; IDR-1018 treated wounds vs. untreated wounds). Moreover, although the rate of wound closure was slower in animals treated with lower doses, even small amounts of IDR-1018 (2 µg/ml) improved wound closure on days 8–10 (Fig 2).

### IDR-1018 induced faster wound closure than previously described wound healing peptides

**Figure 3:**
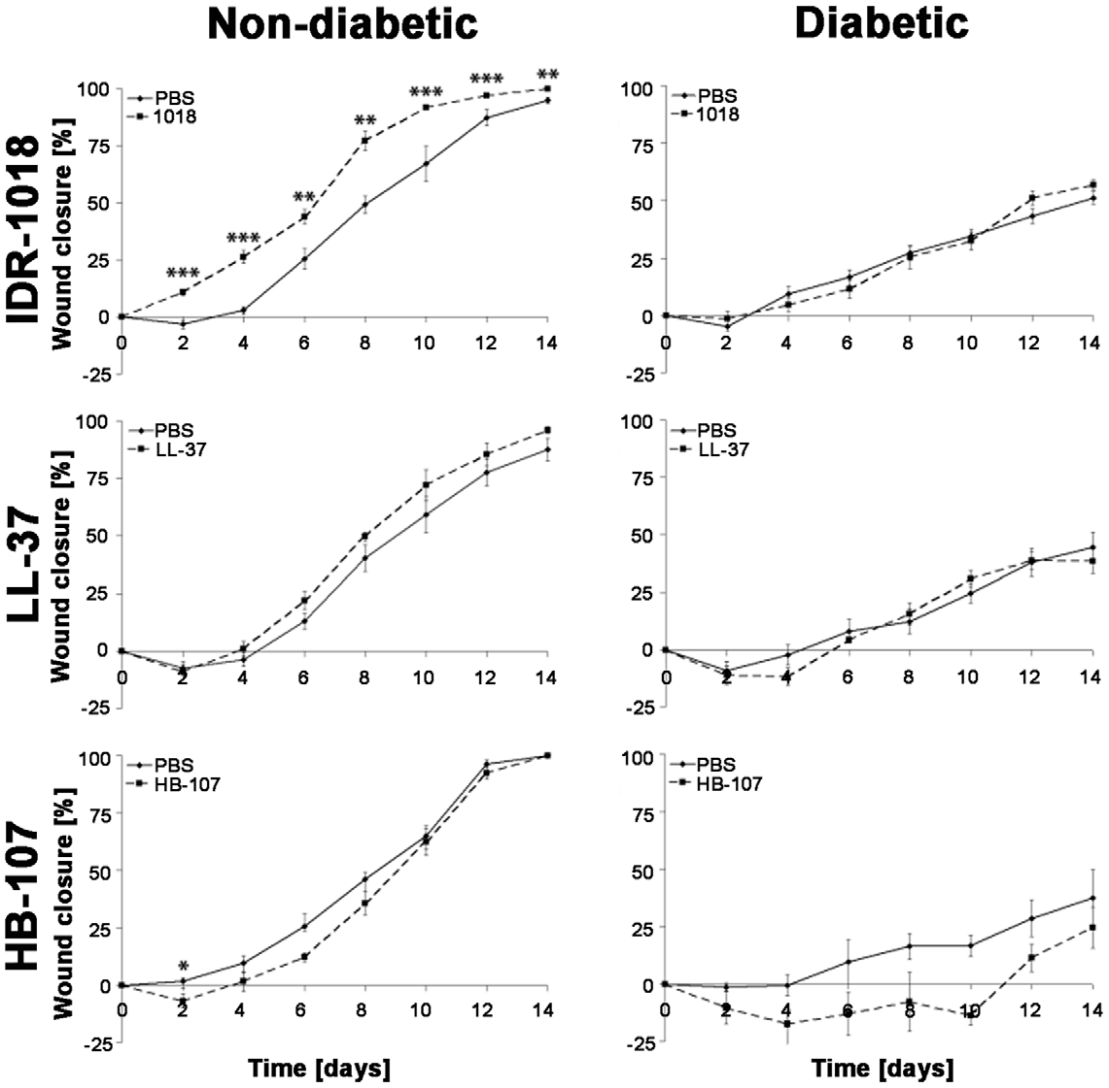
Efficacy in murine wound healing of IDR-1018 compared to LL-37 and HB-107. On day 0, mice were wounded on two dorsolateral areas. Treatments were administered every 48 hours for 14 days with either 15 µl of vehicle control (PBS; wound one) or 200 µg/ml (3 µg/dose) of the indicated peptides (wound two). Re-epithelialization was measured every second day. The bars represent standard error of the mean wound closure (*  = p<0.05; **  = p<0.01; ***  = p<0.005 compared to vehicle control).

To further characterize the wound healing activities of IDR-1018, we compared it to two peptides with previously characterized wound healing activities, the human cathelicidin LL-37 and the synthetic peptide HB-107. At the 200 µg/ml (3 µg/dose) level, treatment of wounds with IDR-1018 led to significantly higher rates of re-epithelialization in non-diabetic mice as compared to LL-37 and HB-107. By day 2, a significantly faster wound closure was measured in IDR-1018 treated wounds compared to untreated wounds (14%, p<0.0001). Further, IDR-1018 treated wounds were 28% smaller than control wounds on day 8 (d8, p = 0.0018). In contrast, neither HB-107 (which led to a slight but non-significant decrease in the rate of wound healing; p = 0.0577–0.4415 from day 2–14; Fig. 3) nor LL-37 (p = 0.1586–0.9243; day 2 14) improved re-epithelialization compared to carrier control treated wounds in diabetic mice. Although there was a tendency towards better re-epithelialization with LL-37 on day 4 (p = 0.1586 respectively) this tendency was lost over time (Fig. 3).

### IDR-1018 showed no effect in diabetic mouse wound healing

Interestingly, the effects of IDR-1018 on experimental diabetic (type-II) wounds contrasted with the positive impact on wound healing in non-diabetic mice. IDR-1018 treated diabetic mice (n = 5) demonstrated a time-course of wound-closure comparable to PBS-treated wounds (±4% compared to PBS-treated wounds, p = 0.1122 to 0.7627, days 0–14). This was also demonstrated for LL-37 as diabetic wounds treated with LL-37 (n = 5) were not significantly different from PBS-treated wounds (±4.6% compared to PBS-treated wounds, p = 0.1586–0.9243; days 0–14). In contrast, HB-107 (n = 5) exhibited a larger, but not significant, wound area compared to control wounds throughout the experiment (day 10: 30% larger wound area in HB-107 treated group, p = 0.057; Fig. 3).

### IDR-1018 in *S. aureus* infected wounds in pigs

We next sought to examine the wound healing activities of IDR-1018 in a different animal species. Using a porcine wound healing model we assessed both anti-infective activity and wound repair function. Full-thickness skin wounds (n = 12 per animal) were made on the backs of two pigs and were subsequently inoculated with *S. aureus*. Wounds were treated with peptide every 48 h and evaluated for wound diameter and bacterial burden over time. We also compared LL-37 and vehicle alone control treatment.

**Figure 4:**
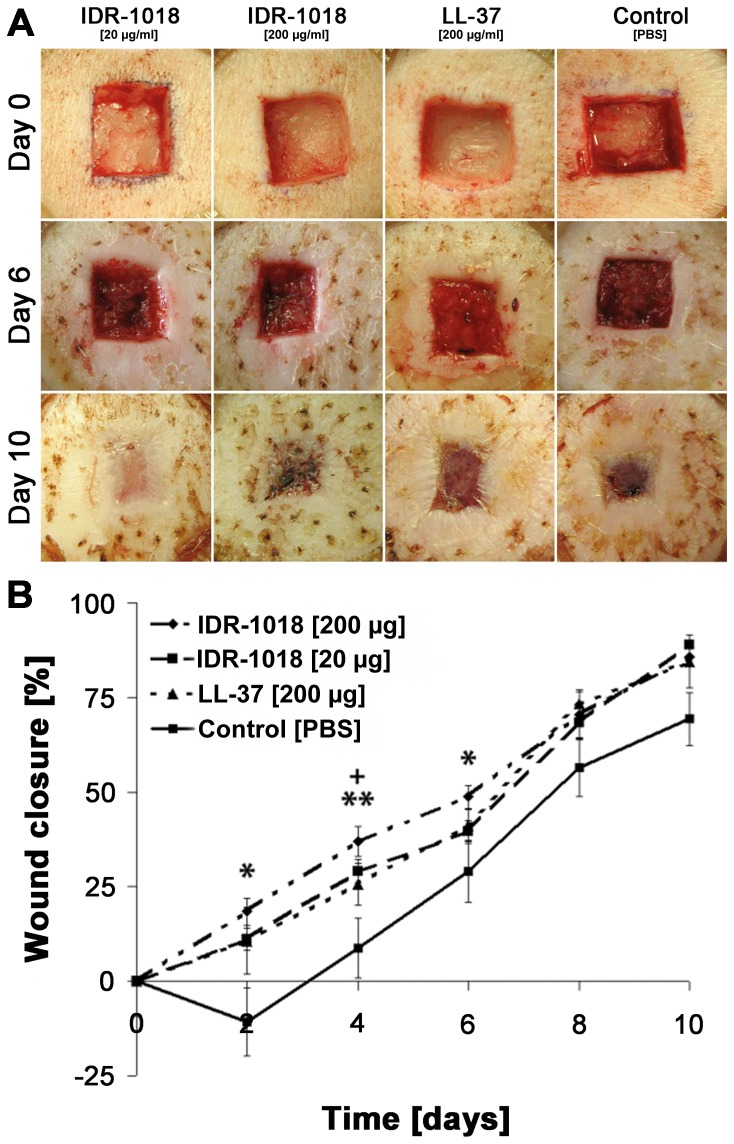
Efficacy in porcine wound healing of IDR-1018 compared to LL-37. (A) Efficacy in an infected porcine model of wound healing by IDR-1018. Digital photographic overview of porcine wounds treated with IDR-1018 (20 or 200 µg/ml every 48 hours) compared to LL-37 (200 µg/ml, positive control) and PBS (carrier control). (B) Quantification of re-epithelialization in wounds treated with IDR-1018. Re- epithelialization was assessed with IDR-1018 (20 and 200 µg/ml), LL-37 (200 µg/ml) or PBS. For day 0 to day 6 each value was calculated as mean out of six wounds while values of day 8 and 10 were calculated out of three different wounds. The bars represent standard error of the mean wound closure (*,+  = p<0.05; **,++  = p<0.01 (*IDR-1018 200 µg/ml; ^+^IDR-1018 20 µg/ml) compared to vehicle control).

On day 1 post-infection, all wounds showed clinical signs of inflammation including swelling and redness (Fig 4a). By day 4 the wound fluid was cloudy and a fibrinous- purulent wound clot formed on top of the wounds. In all groups, representative cross-sectional biopsies were taken on days 6 and 10 after surgery and demonstrated that the high dose of IDR-1018 resulted in almost complete healing by day 10.

IDR-1018 treated wounds demonstrated significantly enhanced wound healing/re-epithelialization as compared to PBS treated wounds (day 4: 28% improvement with a 20 µg/ml IDR-1018; p = 0.0069 and 17% improvement with the 20 µg/ml IDR-1018; p = 0.0289) (Fig 4b). LL-37 (200 µg/ml) treated wounds also showed a higher rate of re-epithelialization as compared to the PBS treated wounds (12–21% faster re-epithelialization on days 2–10, p = 0.0733–0.1982). Epidermal healing in IDR-1018 treated wounds occurred in a dose dependent manner (60–104% greater re-epithelialization of wounds treated with 200 µg/ml IDR-18 compared to 20 µg/ml; p = 0.0319–0.3410). In addition, a significantly higher rate of re-epithelialization was detected in wounds treated with IDR-1018 compared to the same dose of LL-37 (200 µg/ml; days 2–6; p = 0.007–0.03). The highest dose of IDR-1018 led to an enhancement in re-epithelialization of up to 30% compared to PBS treated control wounds. A lower dose of IDR-1018 (20 µg/ml) resulted in a similar pattern of re-epithelialization as compared to the highest dose of LL-37 (200 µg/ml) providing further evidence for the potent wound healing activities of IDR-1018.

**Figure 5:**
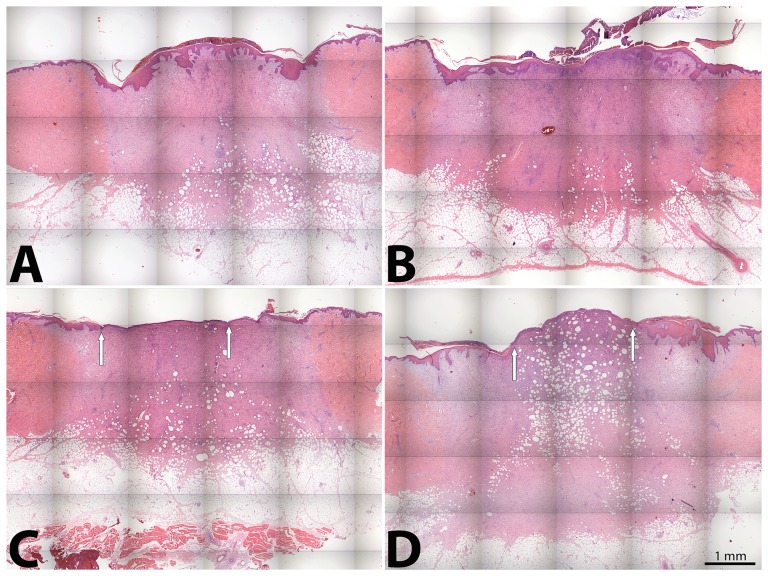
IDR-1018 promoted early keratinocyte proliferation in porcine wounds. Representative examples of paraffin embedded, hematoxylin-eosin-stained sections of porcine wounds at day 10 of the study. Sections show enhanced re-epithelialization in the IDR-1018–treated wounds (A: 200 µg/ml and B: 20 µg/ml) compared to LL-37 (200 µg/ml) (C) and PBS (D) treated wounds at wound edge (white arrow).

Hematoxylin-eosin staining demonstrated that both IDR-1018 and LL-37 evenly formed new epithelium evenly including connective tissue papillae. In contrast, wounds treated with PBS showed decreased organization of newly formed epithelium (Fig 5). IDR-1018 treated wounds exhibited advanced, concentration-dependent, re-epithelialization with keratinocytes migrating from the wound edges towards the centre of the wound forming epithelial tongues. LL-37 treatment resulted in reduced re-epithelialization compared to an equal dosage of IDR-1018.

**Figure 6:**
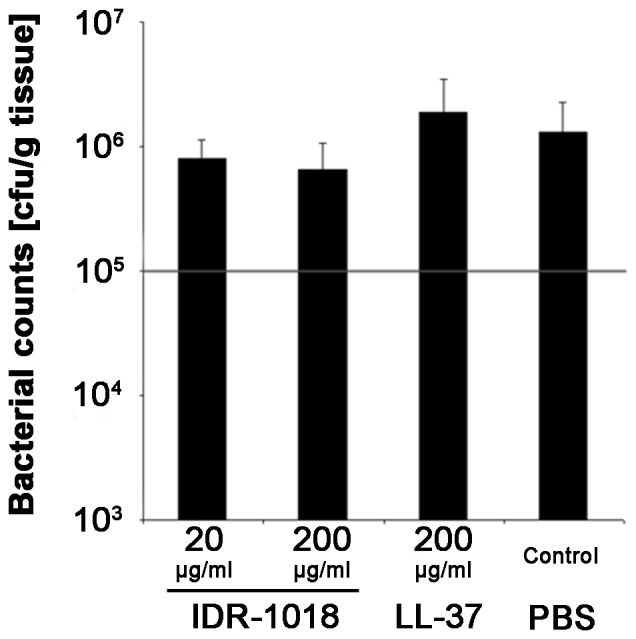
Assessment of bacterial colonization in *S. aureus* infected, porcine skin on day 10 after infection. Wounds were treated with the indicated concentrations of IDR-1018 or LL-37 (positive control). PBS served as negative control. Standard deviation was assessed as SEM.

Interestingly, assessment of bacterial colonization in the wound-tissue showed no significant differences between the treatment groups. On day ten post-infection, bacterial quantification revealed 8×10^5^ cfu (colony forming units)/g tissue for the IDR-1018 20 µg/ml treatment group (n = 3; p = 0.4047 cf. PBS-control) and 7×10^5^ cfu/g tissue for the 200 µg/ml group (n = 3; p = 0.2998 cf. PBS-control). In contrast LL-37 application resulted in a colonization of 1.8×10^6^ cfu/g tissue (n = 3; p = 0.5968 cf. PBS-control) while PBS-control (n = 3) itself had 1.3×10^6^ cfu/g tissue; Fig 6).

## Discussion

In addition to their ability to modulate innate and adaptive immunity, HDPs have also been implicated as positive effectors of wound repair [18], [31], [32], [33]. Recent structure guided investigations of HDPs have focused on identifying the particular sequence motifs responsible for these immunomodulatory activities in an effort to create optimized peptide sequences of minimal sequence length and complexity, the IDRs. Indeed, recently developed optimized IDRs have been demonstrated to elicit strong immunomodulatory activities *in vitro* and *in vivo* and confer protection in murine models of bacterial infection in the absence of significant direct antimicrobial activity [24], [25]. Their mechanism of action largely involves enhanced chemokine production accompanied by suppression of the production of pro-inﬂammatory cytokines, such as TNFα, and is mediated through modulation of specific intracellular signalling pathways.

Peptide based immunotherapies are still in their infancy and there has been a paucity of clinical investigations from which to derive knowledge. This study investigated the new designer peptide IDR-1018 for its ability to influence wound repair. IDR-1018 was selected from a library of scrambled bovine HDP bactenecin derivatives due to its potency in inducing chemokines *in vitro* [29]. Normal wound repair involves the precise orchestration of inflammation, epithelialization, tissue granulation and remodeling. It has been demonstrated that HDPs influence all of these mechanisms lending further credence to our investigations [17], [19], [24]. For example, the cathelicidin PR-39 possesses anti-inflammatory qualities by inhibiting neutrophil oxidase activity, and induces syndecans, heparin sulfate proteoglycans important in wound repair [17], [18]. Another member of the cathelicidin family, the human HDP LL-37, has been demonstrated to suppress pro-inflammatory responses [17] and positively influences the re-epithelialization of human skin wounds [7], [33]. Furthermore, human β-defensin 3 (hBD-3), promotes wound healing in infected porcine diabetic wounds [5]. As these peptides also possess intrinsic antimicrobial activities it has been difficult to discern whether antimicrobial activity is integral to their positive influence on wound repair. Further, therapeutic applications for LL-37 and hBD-3 are limited due to their large size, and thus high associated costs of production, as well as their sequence complexity (hBD-3 disulfide bridges) and high cytotoxicities. In contrast, HB-107, a synthetic HDP derived from the moth antimicrobial cecropin B [34], was found to aid in wound repair by stimulating keratinocyte proliferation and enhancing chemokine-induced leukocyte infiltration despite its lack of antimicrobial activity [21].

In this study we have demonstrated that in addition to its previously reported *in vitro* immunomodulatory activities, IDR-1018 also possesses potent wound healing activities *in vivo*. IDR-1018 elicited stronger wound healing activities in terms of wound closure than either HB-107 and LL-37 in a murine wound healing model. Furthermore, IDR-1018 treatment demonstrated a significantly higher rate of re-epithelialization compared to LL-37 and HB-107 and IDR-1018 showed lower cytotoxicity towards skin cells *in vitro* being significantly less cytotoxic than LL-37 and HB-107, consistent with previous studies with PBMC (peripheral blood mononuclear cells) [29].

A dose response study in a murine splint model demonstrated a dose-dependent effect of IDR-1018 on re-epithelialization. Wound treatment with as little as 30 ng/dose of IDR-1018 was associated with a comparable effect to 100-fold higher doses of LL-37 or HB-107. Higher concentrations of IDR-1018 (0.3 or 3 µg/doses) led to a significantly higher re-epithelialization compared to the same dose of LL-37 or HB-107.

To study the wound healing properties of IDR-1018 in another species and a more complex wound environment, IDR-1018 was tested in a *S. aureus* infected porcine wound model. The data obtained for IDR-1018 indicated it also a dose-dependent effect in re-epithelialization of infected wounds. Histological analysis of IDR-1018 treated wounds revealed a significantly increased re-epithelialization in IDR-1018 treated wounds compared to controls, and indicated that IDR-1018 may influence keratinocyte proliferation or migration.

Quantification of bacterial colonization did not exhibit any significant differences between treatment groups. Together with data from enhanced re-epithelialization in IDR-1018 treated groups of non-diabetic mice and pigs, this finding confirms data about lack of direct anti-microbial and enhanced immunomodulatory activity of IDRs described previously [23], .

Diabetic wounds are associated with a high rate of complications in wound healing independent of infection [35]. Analysis of the wound healing activities of IDR-1018 in diabetic wounds was performed in db/db mice. These db/db mice are leptin receptor deficient and represent a model of type II diabetes mellitus characterized by hyperglycemia, obesity, hyperinsulinemia, and impaired wound healing [36]. Interestingly, data from this study did not demonstrate any effect of IDR-1018 on diabetic wound healing, suggesting the wound healing promoting mechanism is absent or suppressed in diabetic wounds. Previous studies have indicated that diabetes negatively influences immune responses, while IDR peptides work by modulating these responses; thus it is likely that the compromised immune system in diabetic animals blocks one or more signaling pathways by which IDR peptides exert their effects.

In conclusion, we have demonstrated here that in addition to the anti-infective and anti-inflammatory properties demonstrated by IDR peptides, the novel designer peptide IDR-1018 promotes wound healing in two different wound healing models without any significant antibacterial activity in this context.

## Materials and Methods

### Cell culture

Human skin was obtained in the operating room of the BG University Hospital Bergmannsheil. All patients gave their written, informed consent, and the study was approved by the ethics committee of the BG University Hospital Bergmannsheil.

Primary human fibroblasts (HFB) were obtained by incubating small pieces of dermis from human volunteers in 1% collagenase type II (Cell Systems, St. Katharinen, Germany) in PBS for 2 hours with shaking at 37°C. The solution was then filtered through a 100 µm cell strainer, centrifuged and resuspended in DMEM containing 10% fetal bovine serum (FBS) and 1% penicillin/streptomycin (PAA, Coelbe, Germany). Medium was changed every second day. All cell types, including the HaCaT keratinocytes cell line (kindly supplied by Prof. Fusenig, University of Heidelberg) [37], were cultured in DMEM containing 10% FBS and 1% penicillin/streptomycin at 37°C in a humidified atmosphere of 5% CO_2_.

### Peptides

IDR-1018, LL-37 and HB-107 were synthesized by GenScript (Piscataway, NJ, USA) or The Nucleic Acid/Protein Synthesis Unit at UBC using solid phase Fmoc chemistry and purified to a purity >95% using reversed phase HPLC. Peptide mass and lack of contaminating peptides were confirmed by mass spectrometry.

### MTT-Assay

HFB and HaCaT cells were seeded in a 96-well microtiterplate (Omnilab, Bremen, Germany). After 24 hours, peptides were added at the indicated concentrations. Cells were cultured for an additional 24 hours as described above, and 0.5 mg/ml 3-[4,5-dimethylthiazol-2-yl\-2,5-diphenyl tetrazolium bromide (MTT) (Sigma, Taufkirchen, Germany) was added. After four hours 0.01N HCl containing 10% sodium dodecyl sulfate (SDS) was added to stop the reaction, cells were lysed with dimethylsulfoxide (DMSO) and adsorbance at 562nm measured using an ELISA plate reader (BioTek Instruments, Bad Friedrichshall, Germany).

### Murine wound healing model

The protocol utilized complied with all regulations relating to animal use and other federal statutes. It was conducted in compliance with the principles in the ‘Guide for the Care and Use of Laboratory Animals’ from the German Animal Welfare Act. Six week old non-diabetic (C57BLKS) and diabetic (C57BLKS-LepR; leptin receptor deficient; “db/db”) mice (Charles River, Sulzfeld, Germany) were used. Mice were anesthetized by isoflurane inhalation. The dorsal surface was shaved, sterilized with betadine and draped. Two full-thickness wounds, one on each side of midline, were made using a 4 mm punch biopsy created. A donut-shaped splint with a diameter of 1 cm was fashioned from a 0.5 mm-thick silicone sheet (Grace Bio-Laboratories, Bend, USA). An immediate-bonding adhesive was used to fix the splint to the skin followed by interrupted nylon sutures (Ethicon, Inc., Somerville, NJ). Tegaderm (transparent polyurethane bandage) was used as an occlusive wound dressing (Tegaderm, 3M, Neuss, Germany).

Wounds were treated with 15 µl of peptide solution containing 2, 20 or 200 µg/ml (i.e. 30, 300 or 3000 ng) of peptide or PBS as the carrier control every 48 hours (n = 5 animals per group; one treatment and control wound per animal). On the 14th post operative day, mice were euthanized, wounds excised, bisected and stored for histological analysis.

### Porcine wound healing model

Two female Goettinger Minipigs (Ellegaard, Dalmose, Denmark) weighing 20–25 kg at arrival were allowed to acclimatize for 4 weeks prior to initiation of the experiment. Anesthesia was induced with Ketamine/Xylazine (Xyla-Ject, Phoenix, St Josephs, MO, USA) via intramuscular injection. Animals were then transferred to general anaesthesia with 2–3% isoflurane in oxygen (Novaplus, Hospira Inc.). Prior to surgery, the porcine dorsum was shaved and 12 squares measuring 1.5×1.5 cm were outlined using a template and a tattoo gun (Tattoo Age, Dietenheim, Germany). The paraspinal area was disinfected using 10% povidone iodine paint. Twelve full thickness wounds per animal (1.5×1.5×0.5 cm) were created. An adhesive polyurethane chamber (Coloplast, Hamburg, Germany) was placed over each wound.

For bacterial inoculation, a methicillin-sensitive strain of *S. aureus* (ATCC29523) was used. A single colony was inoculated into Laura Bertani culture media (Becton & Dickinson, Heidelberg, Germany), and incubated for 18 hours at 37°C. The culture was centrifuged and resuspended in sterile PBS and adjusted to a final amount of 6.7×10^9^ colony-forming units (cfu)/ml by use of the equation (cfu/ml = OD_600nm_×2.5×10^8^). To immerse the enclosed wound surface, 75 μl of the bacterial suspension was injected into each chamber.

Wounds were treated with 85 µl of peptide solution (n = 3 wounds per group per animal) every 48 hours. On day 6 and 10 post operation, pigs were euthanized, wounds excised, bisected and stored for further analysis.

### Histology

Samples from each experiment were fixed in 4% buffered paraformaldehyde. Tissue samples were stained with haematoxylin/eosin, analyzed by three independent investigators. Images were taken with an AxioCam camera on an Axioplan microscope (Carl Zeiss GmbH, Oberkochen, Germany).

### Wound analysis

Digital photographs were taken every second day. Wound closure was calculated by measuring the wound area from the computerized image with Photoshop CS4 Pro software (Adobe, San Jose, USA) using the formula (Wound area)/(Inner area of polyurethane chambers or silicon splints). Wound area was calculated as the percent area of the original wound.

### Assessment of wound infection

Tissue samples were homogenized in PBS (PT3100 Polytron, Kinematica, Littau-Luzern, Switzerland). Serially diluted aliquots of homogenate were quantified on Luria–Bertani agar (IDC, Bury, Lancashire, UK) after incubation for 18 h at 37°C. cfu/g tissue was calculated as described previously [38]. A stable wound in fection was defined as 10^5^ colony forming units (cfu)/g tissue.

### Statistical analysis

Data were analyzed using analysis of variance and independent sample t-test with the software package software package SPSS 14.0 (SPSS, Chicago, IL, USA). A p-value of less than 0.05 was considered significant.
